# Costoclavicular Brachial Plexus Block for Upper Limb Procedures: A Narrative Review of Efficacy and Safety

**DOI:** 10.7759/cureus.104981

**Published:** 2026-03-10

**Authors:** Richa Kewalramani, Anuradha Vaswani, Khushboo Bairwa, Rajashree Munavalli, Sushil K Bhati

**Affiliations:** 1 Department of Anesthesiology and Critical Care, All India Institute of Medical Sciences, Jodhpur, Jodhpur, IND; 2 Department of Anesthesiology, Kshetrapal Multispeciality Hospital and Research Centre, Ajmer, IND; 3 Department of Anesthesiology and Critical Care, PES University-Institute of Medical Sciences and Research (IMSR), Bangalore, IND; 4 Department of Anaesthesiology, Sawai Man Singh Medical College, Jaipur, IND

**Keywords:** costoclavicular block, diaphragm-sparing anaesthesia, infraclavicular block, regional anaesthesia, upper limb surgery

## Abstract

Regional anesthesia techniques for upper limb surgery continue to evolve to improve block reliability while minimizing approach-specific complications. This narrative review evaluates the efficacy and safety of the costoclavicular brachial plexus block, a modified infraclavicular approach that targets the clustered cords of the brachial plexus lateral to the axillary artery. The objective is to synthesize current evidence on clinical performance, analgesic outcomes, and complications in upper limb procedures. A structured literature review of studies published between 2015 and 2025 was conducted using major medical databases, including randomized trials, observational studies, systematic reviews, and relevant anatomical investigations. The evidence demonstrates that the costoclavicular block provides reliable surgical anesthesia and postoperative analgesia for distal and intermediate upper limb procedures, with high success rates and predictable onset under ultrasound guidance. Continuous catheter techniques further extend its analgesic utility. A consistent and clinically significant finding is the lower incidence of hemidiaphragmatic paralysis compared with interscalene and supraclavicular blocks, supporting its use in patients with limited respiratory reserve. Complication rates, including vascular puncture and neurological injury, are low and comparable to other infraclavicular techniques. Although the block alone may not provide complete anesthesia for extensive shoulder surgery, it offers a safe, efficient, and diaphragm-sparing alternative for a wide range of upper limb procedures. This review supports the costoclavicular approach as an important component of contemporary, anatomy-based regional anesthesia practice.

## Introduction and background

Regional anesthesia is a key factor in modern perioperative care of upper limb surgery, as it offers stable surgical anesthesia, better postoperative pain management, and a significant decrease in opioid use [1]. The development of brachial plexus block methods has been constantly supported by the dual goals of enhancing the reliability of the block and reducing complications based on approach [2]. The development of ultrasound technology has also increased this development by providing real-time visualization of neural and non-neural structures, greatly increasing efficacy and safety [3].

The protocols of brachial plexus block established are each accompanied by their own benefits and limitations [4]. The interscalene block is the procedure of choice in shoulder surgery and is always linked with a high rate of hemidiaphragmatic paralysis and decreased distal coverage [5]. Supraclavicular block gives dense anesthesia in most of the upper limb, but the fear of phrenic nerve involvement and pneumothorax continues, especially in patients with impaired pulmonary function [6]. Axillary block does not produce diaphragmatic dysfunction and is often associated with the need for multiple injections and may lead to incomplete anesthesia of proximal surgeries [7]. Some of these limitations are addressed with traditional infraclavicular methods, but the techniques may prove to be difficult to work with due to the deep nature of the needle pathways, the variable position of the cords, and the proximity to the great supporters [8].

The costoclavicular brachial plexus block has become an advanced position in the intra-clavicle approach that builds on the reliability of the anatomical relationships in the costoclavicular approach [9]. The lateral, posterior, and medial cords of the brachial plexus are clustered to the lateral of the axillary artery beneath the clavicle at this level [10]. This foreseeable positioning will make the needle track smoother, better viewed on ultrasound, and equal distribution of local anesthesia, which can be performed in a single injection [11]. These characteristics are a conceptual change to anatomy-based accuracy instead of dependence on larger fascial compartment expansion [12,13].

Costoclavicular brachial plexus block is basically one of the types of infraclavicular blocks. It is a reliable technique due to its ease of access to the cords of the brachial plexus, which are situated lateral to the axillary artery in the costoclavicular space [14]. The dynamic studies of block dynamics by ultrasound have shown that circumferential dispersion of local anesthetic around the clustered cords is rapid and associated with a short onset time and high success rates of a block [15]. Technical changes have also increased reproducibility between operators with different levels of experience, such as differences in needle entry point and injection strategy [16].

**Figure 1:**
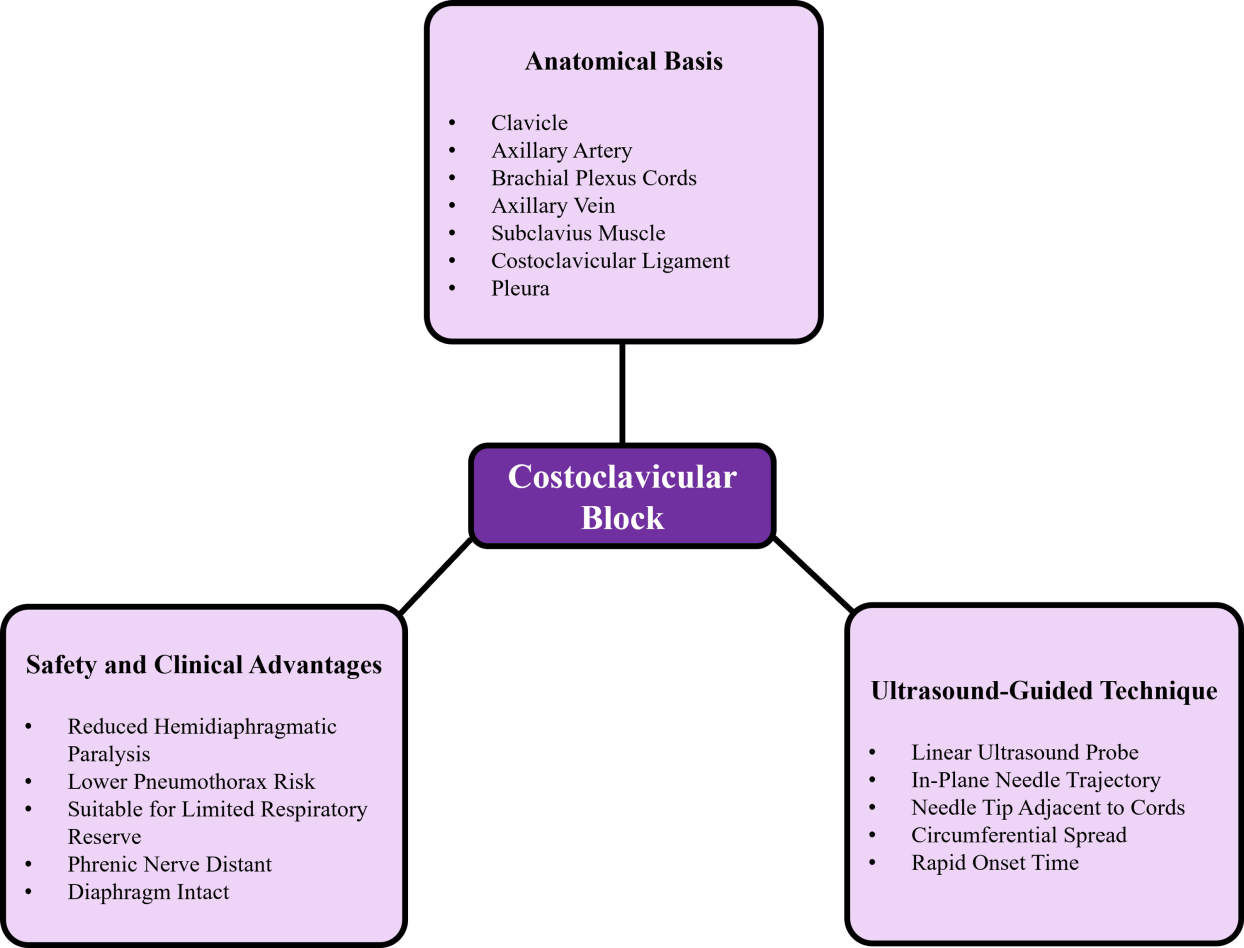
Conceptual overview of the costoclavicular brachial plexus block Created by the authors using Microsoft PowerPoint (Microsoft Corporation, Redmond, WA, US)

The rising popularity of the costoclavicular approach has been characterized by safety concerns [17]. The costoclavicular block seems to be linked with a lower rate of phrenic nerve palsy as compared to supraclavicular and interscalene methods, and this benefit is of special importance in patients with a limited respiratory reserve [18]. The lateralized needle pathway and the greater distance between the pleura and the needle could also act as a mitigant against the risk of pneumothorax [19]. Evidence-based cadaveric experiments and imaging analysis helped establish the spatial arrangements of vital structures in the needle path, and the safety profile of this method is supported when the ultrasound guidance is applied [20]. Figure 1 illustrates the anatomical relationships within the costoclavicular space relevant to the block technique.

The available literature includes randomized trials, systematic reviews, meta-analyses, cadaveric studies, and case reports of both adults and children. In spite of this increasing evidence, there are weaknesses in terms of interpretation due to heterogeneity in terms of study design, outcome definitions, local anesthetic regimens, and technical variations. Lacking consistency in nomenclature and reporting of inconsistent outcomes of complications also complicates cross-study comparisons and generalizability of clinical studies. A focused narrative review examining upper limb procedures is therefore warranted.

Objectives of the Review

This narrative review provides a summary of existing evidence on the effectiveness of the costoclavicular brachial plexus block in upper limb surgery. It critically evaluates safety outcomes and profiles of complications related to this method and compares its performance to other established methods of brachial plexus block. It also determines knowledge gaps available and outlines future research priorities.

Methodology

Technique Description

A standardized description of the costoclavicular brachial plexus block, reflecting the technique most commonly employed across the included studies, has been incorporated. In most reports, the block is performed under ultrasound guidance with the patient in the supine position, the arm abducted, and the transducer placed inferior and parallel to the clavicle to identify the clustered cords lateral to the axillary artery within the costoclavicular space, followed by in-plane needle advancement and deposition of local anesthetic around the cords.

Literature Search Strategy

This narrative review was conducted in accordance with established principles for non-systematic evidence synthesis. A comprehensive literature search was performed for studies published between January 2015 and December 2025. Electronic databases searched included PubMed/MEDLINE, Embase, Scopus, Web of Science, and the Cochrane Library. Additional references were identified through manual screening of the bibliographies of relevant articles.

Study Selection Process

Duplicate records were removed prior to screening. Title and abstract screening was followed by full-text assessment for eligibility, and study selection was performed by two independent reviewers. Disagreements were resolved through discussion and consensus.

Search Terms and Keywords

The search strategy combined controlled vocabulary and free-text terms using Boolean operators. Core search strings included combinations of: “costoclavicular brachial plexus block” OR “costoclavicular block”, AND “upper limb surgery” OR “upper extremity surgery”, AND “regional anaesthesia” OR “ultrasound-guided block”. Boolean operators (AND, OR) were applied to refine sensitivity and specificity, and database-specific filters were used where appropriate.

Eligibility Criteria

Eligible studies included randomized controlled trials, observational studies, systematic reviews, meta-analyses, cadaveric studies, and clinically relevant case reports assessing the costoclavicular brachial plexus block during upper limb surgery in adults or children. Studies were excluded if they evaluated only non-costoclavicular techniques, addressed conditions unrelated to the upper limb, or failed to report clinical outcomes. Only articles published in English were included.

Evidence Appraisal

Given the narrative design and heterogeneity of included study types, formal risk-of-bias scoring tools were not applied. Instead, methodological quality was appraised descriptively based on study design hierarchy, clarity of outcome reporting, internal consistency, and relevance to clinical practice, with greater interpretive weight assigned to randomised trials and meta-analyses. No meta-analysis, meta-regression, or pooled quantitative synthesis was performed due to variability in study design, outcome definitions, and reporting across the included literature.

## Review

Anatomical basis of the costoclavicular space

The costoclavicular space is an easily identifiable infraclavicular area in which the brachial plexus cords take a compact and consistent position [21]. The lateral, posterior, and medial cords are often gathered to the lateral of the axillary artery at this level and are fairly superficial relative to more distal infrastructural positions [22]. This standard anatomical support enables the differentiation of the costoclavicular zone from other sites of the brachial plexus block and forms the structural foundation of the dependable neural targeting [7].

The axillary artery is the main landmark in the costoclavicular space, in which the cords are closely apposed about the lateral side of the axillary artery [23]. In the costoclavicular space, the axillary vein is typically situated anteroinferior to the axillary artery rather than between the arterial structures when the arm is positioned alongside the body. Its relative position may vary with patient positioning and respiratory phase; therefore, the cited reference has been rechecked to ensure anatomical accuracy [14]. The subclavius muscle and costoclavicular ligament are anatomical barrier that separates the pleura and the plexus [24]. Such separation helps provide a comparatively higher margin of safety as compared to supraclavicular ones, but proper control of the needle is necessary due to the proximity of the major vascular structures [9].

**Figure 2:**
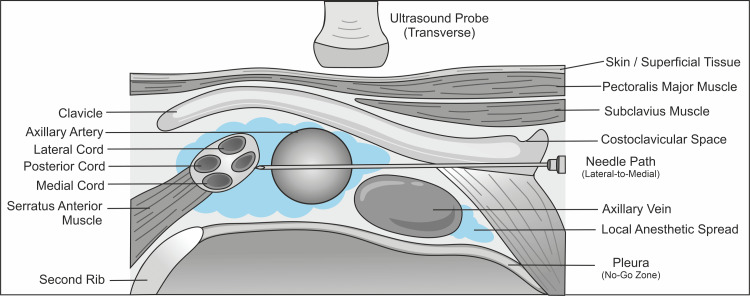
Anatomical and ultrasound-guided orientation of the costoclavicular brachial plexus block Created by the authors using CorelDRAW (Corel Corporation, Ontario, Canada)

The limited range of the fascia of the costoclavicular space guides the access of needles as well as the distribution of local anesthetics [25]. The tight cord configuration can be easily blocked by injection of specific compartments in the anatomy [11]. In comparison to the traditional infraclavicular techniques in which the cords could be separated with the use of multiple deposits, the costoclavicular structure allows uniform circumferential dissemination with a decrease in the number of needle redirections [15]. This anatomical effectiveness provides the technical excellence and repeatability of the technique of the costoclavicular brachial plexus block when administered under ultrasound control [2]. A schematic representation of the transverse sonoanatomy and the lateral-to-medial needle pathway in the costoclavicular space is shown in Figure 2.

Sonoanatomy and technical considerations

Correct localization of sonoanatomical landmarks is mandatory to achieve the performance of the costoclavicular brachial plexus block [26]. A high-frequency linear ultrasound transducer is placed inferior and parallel to the clavicle of the body to see the axillary artery and other brachial plexus cords [27]. The cords usually show up as hyperechoic oval-shaped structures lateral to the artery, and the superficial boundary of the imaging field is the subclavius muscle [8]. The pleura is seen further and in the middle, further stressing the need to keep the depth orientation on as the needle advances [14].

Several needle paths have been outlined, with the most used path being the lateral-to-medial [28]. This path can visualize the needle in-plane and keep a safe distance between it and the pleura, with the least impact on vascular structures [9]. Lateral-to-medial approaches can be applied selectively and need greater attention because they are even more proximate to the axillary vein [29]. Sagittal approaches, where the needle trajectory is nearly perpendicular, could minimize tissue penetration at the expense of close observation of the needle tip in the deeper planes to prevent loss of control [30].

The arrangement of the cords is compact, and it determines the injection strategy [22]. Single-injection methods entail locally depositing local anesthetic around the group of cords, and it is based on circumferential diffusion within the limited space [11]. A multiple-injection technique is used to attack single cords and can be used in the case of anatomical variation or stuttering development [4]. There is comparative evidence that with less time to carry out each procedure and fewer needle manipulations, single-injection procedures are as successful [29]. With or without the methodology, constant ultrasound imaging and gradual injection with frequent aspiration are necessary to reduce vascular puncture and intravascular injection [31].

Block dynamics and mechanism of action

The costoclavicular approach has a block dynamics that is characterized by the rapid and predictable spread of local anesthetic in a confined anatomical compartment [22]. Ultrasound studies have continuously shown that the injectate is circumferentially distributed around the clustered cords, leading to simultaneous blockage of various terminal nerve distributions [11]. This spread pattern increases the rapid onset of sensory and motor blockade, which is attributed to the high reliability reported in clinical studies [15].

The close proximity lowers the diffusion distance and increases the exposure of the nerve to local anesthetic [8]. Consequently, the lower volumes of injection may often be used with great success as compared to the volumes of injections used with conventional infraclavicular techniques that are deemed to be effective [24]. Both efficiency and safety implications of this property exist, specifically in minimizing the cumulative local anesthetic dose [3]. Block onset and completeness variability seem to be lower than methods based on diffusing through less restricted fascial planes [29].

The concentration and volume of local anesthetic have an effect on the balance between postoperative analgesia and surgical anesthesia [4]. Greater levels are related to deep motor and sensory blockage to the use under surgical conditions, and less under the circumstances of less severe analgesia with partial retention of motor activity [32]. These parameters can be further fine-tuned due to the foreseeable distribution and limited anatomy of the costoclavicular space [10]. These features justify the increasing popularity of the costoclavicular method of single-injection anesthesia as well as of the continuous catheter method [18].

Efficacy in upper limb surgical procedures

Costoclavicular brachial plexus block has proven to be uniformly effective in a broad spectrum of surgical operations on the upper limbs, and this has been corroborated by randomized trials and comparative clinical trials [31]. Its most familiar use is in surgery of the forearm, wrist, and hand, where there is a need to anesthesise with reliability the median, ulnar, radial, and musculocutaneous nerves [12]. In several studies, the costoclavicular method demonstrates excellent success rates of a complete sensory and motor block, where intraoperative supplementation or general to general anesthesia conversion is minimal or absent [32]. The small cord position at the costoclavicular level also helps in predictable neural coverage and minimizes interpatient variability of block success [21].

It has also been shown that the costoclavicular block is effective for the elbow and humeral procedure [33]. Comparative studies reveal non-inferior block success, compared to paracoracoid infraclavicular and supraclavicular usage in an elbow surgery [14]. The costoclavicular block is effective as a consistent anesthetic method used in humeral surgeries, especially at the distal and mid-shaft level, with local anesthetic doses taken care of [22]. It has been more selective in its application in shoulder surgery [11]. Costoclavicular block is not a reliable anesthetic agent for all shoulder innervations, and clinical trials indicate that it can be used as an alternative or supplement to the interscalene block, especially in patients who are prone to respiratory compromise [24]. In this regard, it helps in successful analgesia without routine phrenic nerve blockage [25].

Comparative data also explain the clinical positioning of the costoclavicular approach [34]. Compared with the supraclavicular block, it provides a similar anesthetic effect for distal upper limb surgery while being associated with a lower incidence of hemidiaphragmatic paralysis [23]. The costoclavicular approach provides better musculocutaneous nerve coverage and does not require multiple distal injections, leading to shorter performance times and increased efficiency of the procedure as compared to the axillary block [15]. Costoclavicular block has been demonstrated to be equally successful with a reduced number of needle passes and earlier onset when compared with the more traditional infraclavicular methods in several studies [13]. These results endorse the costoclavicular block as a technically effective and clinically useful alternative in a variety of procedures on the upper limb [16]. Table 1 shows that the costoclavicular brachial plexus block provides reliable and versatile anesthesia across a range of upper limb procedures.

**Table 1:** Clinical performance of the costoclavicular brachial plexus block Compiled by the authors based on information synthesized from [11,13-16,21-24,31-33].

Surgical Region	Comparator Technique	Key Clinical Outcome	Clinical Implication	Reference
Forearm, wrist, and hand surgery	—	Reliable anesthesia of the median, ulnar, radial, and musculocutaneous nerves	Suitable primary regional technique for distal upper limb surgery	[31]
Forearm, wrist, and hand surgery	—	High rates of complete sensory and motor blockade	Minimal need for intraoperative supplementation or conversion to general anesthesia	[32]
General block performance	—	Compact cord arrangement enables predictable neural coverage	Reduced interpatient variability in block success	[21]
Elbow surgery	Paracoracoid infraclavicular block	Non-inferior block success	Effective alternative infraclavicular approach	[33]
Elbow surgery	Supraclavicular block	Comparable anesthetic efficacy	Equivalent option for elbow procedures	[14]
Humeral surgery (distal and mid-shaft)	—	Consistent surgical anesthesia with appropriate dosing	Reliable option for selected humeral procedures	[22]
Shoulder surgery	Interscalene block	Incomplete coverage as sole technique	Best used as an adjunct or alternative in selected cases	[11]
Shoulder surgery in high-risk patients	Interscalene block	Effective analgesia without routine phrenic nerve palsy	Advantageous in patients with respiratory compromise	[24]
Distal upper limb surgery	Supraclavicular block	Similar anesthetic efficacy with less diaphragmatic involvement	Respiratory-sparing alternative	[23]
Distal upper limb surgery	Axillary block	Improved musculocutaneous nerve coverage	Eliminates the need for multiple distal injections	[15]
Infraclavicular approaches	Traditional infraclavicular block	Similar success with fewer needle passes and faster onset	Greater procedural efficiency	[13]
Overall clinical application	Multiple techniques	Consistently effective across procedures	Technically efficient and clinically versatile block	[16]

Analgesic quality and duration

The costoclavicular brachial plexus block provides reliable intraoperative anesthesia and quality postoperative analgesia for upper limb surgery [35]. Dense sensory blockade intraoperatively is obtained with a predictable time-of onset, which allows timely surgical conditions [18]. The block can be modified to the surgical requirements to avoid unnecessary prolongation of motor blockade, so there is a possibility of balanced anesthesia according to the needs of the procedure [22]. The costoclavicular approach has a strength in the form of postoperative analgesic outcomes [36]. Clinical trials indicate always lower levels of pain scores and decreased use of opioids during the early postoperative period with this method than with general anesthesia without any or with regional methods that are not as comprehensive [24]. These analgesic effects are most evident after forearm, wrist, and hand surgery, during which a long-term sensory blockade helps in successful pain management during the early phase of recovery [16].

The dosing of local anesthesia is centrally involved in the determination of the analgesic time and motor recovery [19]. Dose-finding and concentration comparison evidence suggests that moderate amounts serve as an effective agent in analgesia, a process that is efficient in terms of local anesthetic dissemination in the confined costoclavicular space [37]. The decreased concentrations permit the maintenance of motor activity without the loss of analgesia and can justify early ambulatory and ambulatory surgery, which is appropriate [25]. On the other hand, higher concentrations are able to give deep anesthesia to larger surgeries without affecting block reliability [20].

**Figure 3:**
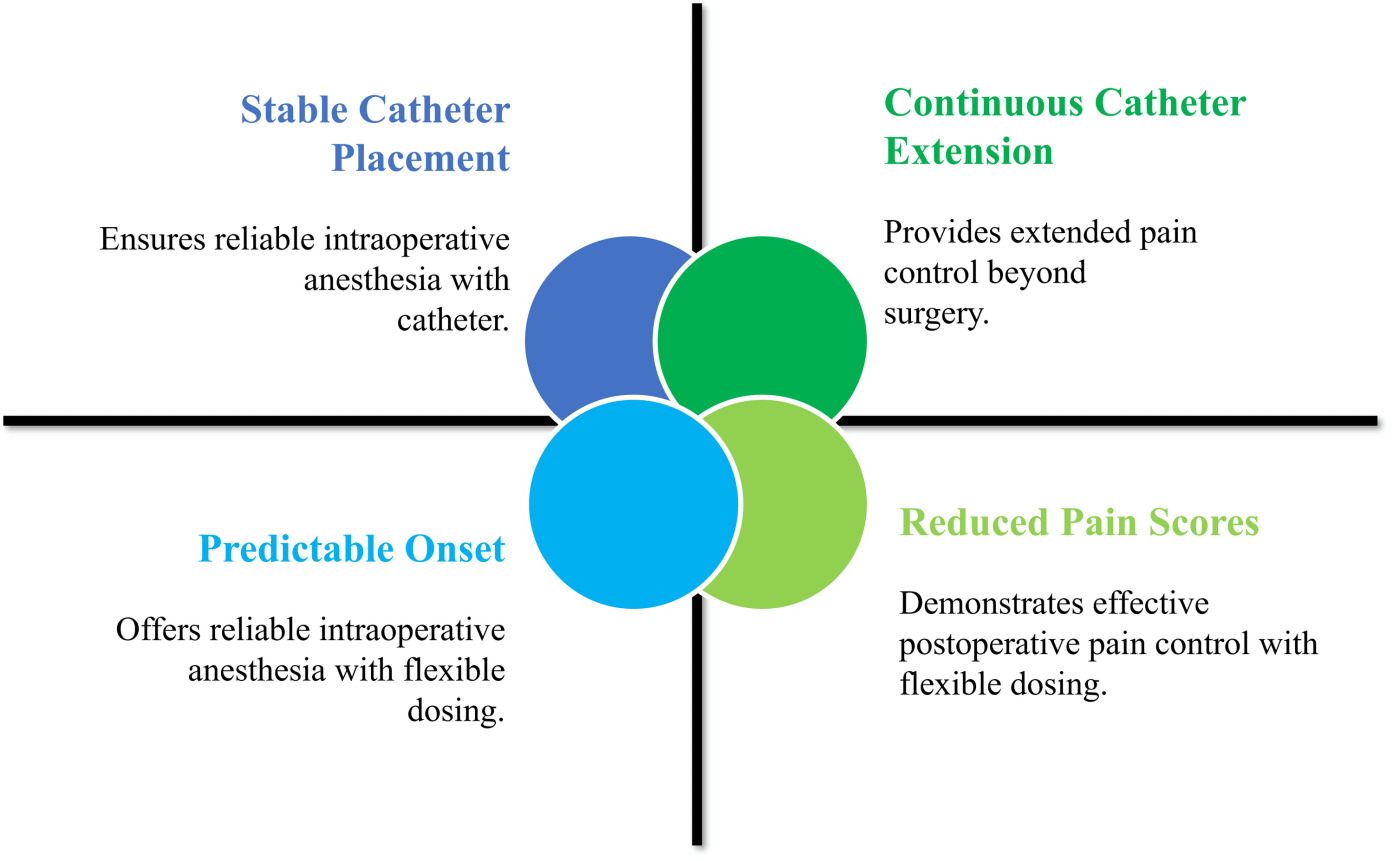
Analgesic profile of the costoclavicular brachial plexus block Created by the authors using Microsoft PowerPoint (Microsoft Corporation, Redmond, WA, US)

The use of continuous catheter placement in the costoclavicular space prolongs the analgesic effects of the method [38]. In clinical reports, stable catheter placement and successful prolonged analgesia after complicated upper limb surgery, which encompasses fractures and reconstructive operations, are exhibited [30]. Costoclavicular catheters can be better tolerated by patients and cause less disturbance of the neck movements compared to more proximal sites of catheter placement [17]. These features make the costoclavicular block a multifunctional analgesic procedure in terms of single-injection and constant-rate analgesic methods [21]. Figure 3 illustrates that the costoclavicular block provides a predictable onset, effective analgesia, and seamless extension with continuous catheter techniques.

Safety profile and complications

The costoclavicular brachial plexus block has a favourable safety profile when performed under ultrasound guidance [39]. As the costoclavicular space is highly vascular, routine use of colour Doppler to identify the axillary artery and vein before needle advancement and injection is recommended as an additional safety measure to reduce the risk of inadvertent vascular puncture [21]. Vascular puncture remains a potential complication due to the close proximity of the axillary vessels, although reported rates are low and comparable to other infraclavicular approaches [27]. Continuous needle tip visualization, incremental injection, and frequent aspiration remain essential procedural precautions [21].

There is limited literature specifically addressing local anesthetic systemic toxicity [40]. Achieving effective blockade with lower local anesthetic volumes may reduce systemic exposure; however, strict adherence to recommended dose limits remains necessary [32]. The incidence of neurological complications, such as transient paraesthesia or neuropraxia, appears low and comparable to other ultrasound-guided brachial plexus blocks [24]. No evidence currently suggests that the risk of persistent nerve injury is higher with the costoclavicular approach [41].

Respiratory complications remain an important consideration in upper limb regional anesthesia [42]. Compared with interscalene and supraclavicular blocks, the costoclavicular approach is associated with a lower incidence of hemidiaphragmatic paralysis, consistent with its greater anatomical distance from the phrenic nerve [43]. Pneumothorax is considered uncommon, likely due to the lateral needle trajectory and the ability to visualise the pleura under ultrasound guidance [28]. Overall, the available evidence supports the costoclavicular brachial plexus block as a safe and effective technique, with complication rates that are similar to or lower than those reported for other brachial plexus block approaches [44].

Respiratory effects and phrenic nerve involvement

Respiratory impairment due to phrenic nerve involvement remains a major limitation of proximal brachial plexus blocks [45]. The phrenic nerve originates from the C3-C5 roots and descends bilaterally along the anterior surface of the anterior scalene muscle before entering the thorax. An interscalene block is performed at the level of the roots and trunks in close proximity to this course and is associated with hemidiaphragmatic paralysis in nearly 100% of cases. The supraclavicular block, performed at the level of the trunks and divisions, is anatomically slightly more distal but still sufficiently close to allow cranial spread, with reported hemidiaphragmatic paralysis rates of approximately 30-60% despite ultrasound guidance and lower local anesthetic volumes [9]. In contrast, the costoclavicular block is undertaken at a more distal infraclavicular level, anatomically separated from the cervical trajectory of the phrenic nerve, and reported rates of hemidiaphragmatic paralysis are generally below 5% in most series [46].

Comparative studies and meta-analyses demonstrate a significantly lower incidence of hemidiaphragmatic paralysis with the costoclavicular technique compared with the interscalene block [47]. Ultrasonographic and pulmonary function assessments show preserved diaphragmatic excursion with minimal changes in forced vital capacity and forced expiratory volume [39]. Compared with the supraclavicular block, the costoclavicular approach provides more consistent diaphragmatic preservation, particularly with moderate local anesthetic volumes, likely due to limited cranial spread toward the cervical plexus [48,21].

This diaphragm-sparing profile has important clinical implications [49]. Patients with chronic obstructive pulmonary disease, restrictive lung disease, obesity-related hypoventilation, or contralateral diaphragmatic dysfunction are at increased risk of respiratory compromise after proximal blocks [13]. The costoclavicular approach allows effective upper limb anesthesia while maintaining respiratory stability in such populations, as supported by randomized trials in proximal humeral and selected shoulder procedures without clinically significant respiratory depression [40]. These findings support its role as a diaphragm-sparing regional anesthesia technique [18]. Table 2 shows that the costoclavicular brachial plexus block preserves diaphragmatic function while maintaining effective upper limb anesthesia.

**Table 2:** Respiratory safety and diaphragm-sparing profile of the costoclavicular brachial plexus block Compiled by the authors based on information synthesised from [9,13,18,21,39,40,45-48,50]. COPD: Chronic Obstructive Pulmonary Disease, FVC: Forced Vital Capacity, FEV: Forced Expiratory Volume

Aspect	Comparator	Key Observation	Clinical Relevance	Reference
Respiratory limitation of proximal blocks	Proximal brachial plexus techniques	Phrenic nerve blockade is a major cause of respiratory impairment	Limits the use in patients with reduced pulmonary reserve	[45]
Interscalene block	—	Hemidiaphragmatic paralysis occurs consistently.	High risk of diaphragmatic dysfunction	[9]
Supraclavicular block	—	Clinically significant diaphragmatic paralysis persists despite ultrasound guidance.	Respiratory risk remains relevant	[9]
Costoclavicular block anatomy	Distal infraclavicular level	Block performed distant from the cervical course of the phrenic nerve	Explains the low incidence of diaphragmatic dysfunction	[46]
Costoclavicular vs interscalene block	Comparative studies and meta-analyses	Significantly reduced rate of hemidiaphragmatic paralysis	Supports the diaphragm-sparing advantage	[47]
Diaphragmatic function assessment	Ultrasound and pulmonary function testing	Preserved diaphragmatic excursion with minimal FVC and FEV changes	Confirms respiratory safety	[39]
Costoclavicular vs supraclavicular block	Moderate local anesthetic volumes	More consistent diaphragmatic preservation	Predictable respiratory outcomes	[48]
Mechanism of diaphragm sparing	Local anesthetic spread	Limited cranial spread toward the cervical plexus	Anatomical basis for reduced phrenic involvement	[21]
High-risk respiratory populations	COPD, restrictive lung disease, obesity hypoventilation	Increased susceptibility to respiratory compromise	Need for diaphragm-sparing techniques	[13]
Costoclavicular block in high-risk patients	Upper limb anesthesia and analgesia	Effective analgesia without respiratory instability	Suitable alternative to proximal blocks	[50]
Proximal humeral and shoulder surgery	Monitored anesthesia care	Adequate analgesia without significant respiratory depression	Expands clinical applicability	[40]
Overall clinical positioning	Diaphragm-sparing strategies	Feasible and effective regional anesthesia option	Integrates into respiratory-protective protocols	[18]

Special populations

The anatomical and technical aspects of the costoclavicular brachial plexus block are useful in several distinct groups of patients [20]. The supraclavicular and axillary approaches may be complicated by an increase in tissue depth and the displacement of the surface landmarks in obese patients [47]. On the other hand, the costoclavicular space is usually available in high-frequency ultrasound because the space is relatively superficial and has a relatively fixed anatomical pattern [21]. The rates of block success, onset, and complication are similar between obese and non-obese patients, which attests to the effectiveness of the costoclavicular procedure in obese patients [41].

The use of the costoclavicular block as a pediatric technique has grown in the use of ultrasound-guided regional anesthesia [17]. Research on children receiving distal and intermediate upper limb surgery also proves effective anesthesia as well as low rates of complications [49]. This is due to the clustered cord anatomy that can be targeted with little needle manipulation, thus making the procedure less complex [22]. Notably, phrenic nerve rates are reportedly low in pediatric patients undergoing costoclavicular block, which is also beneficial considering the fact that there is limited respiratory capacity among the younger patients [9]. Although good results have been observed, caution and compliance with pediatrically specific safety principles are of paramount importance [38].

There is little evidence on use in pregnancy, but it is clinically relevant [6]. The case reports and small series report the successful use of single-injection and continuous use of costoclavicular block in the upper limb trauma and orthopedic surgery in pregnant patients [50]. The method provides effective analgesia but with less exposure to systemic opioids and with no respiratory compromise that is desirable in this context [24]. Pregnancy induces physiological alterations that require conservative dosing, strict observation, and wider inferences within the context of larger prospective studies will be possible [29].

The costoclavicular block has also been found useful in the emergency and non-operating room setting [15]. It is predictable, has a predictable neural coverage, and a safety profile in ultrasound-guided operations, which is why it is applicable in the reduction of acute fractures, joint manipulation, and vascular access operations [10]. The procedure offers effective procedural anesthesia in emergency department settings without deep sedation or airway instrumentation, and promotes effective and patient-centred care [32].

Comparative evidence and clinical positioning

The clinical role of the costoclavicular brachial plexus block as compared to conventional methods is determined through comparative evidence provided by the randomised controlled trials and meta-analyses [34]. Costoclavicular block proves to have the same success rates as that of supraclavicular and paracoracoid infraclavicle blocks in distal and intermediate upper limb surgery [31]. Other studies indicate the same or earlier onset and fewer needle passes, which indicate positive sonoanatomy and efficient local anesthetic distribution [33].

The unique benefits of the costoclavicular technique are reported to be the ability to see the cord reproducibly, decreased depth of the needle, and consistent block dynamics [37]. A clinically significant advantage of lower hemidiaphragmatic paralysis rates than interscalene and supraclavicular blocks is specifically in respiratorily vulnerable patients [39]. Also, a moderate amount of local anesthetic may frequently be effective in preventing blockade and undergoing, and is safe since it does not compromise the procedure [32].

The constraints of the costoclavicular block need to be identified [38]. In surgeries where complete shoulder anesthesia is necessary, the technique in most cases is not enough and might need a supplement or other methods [45]. The experience of the operator is known to have effects on the performance of the block, especially in the early adoption, because the costoclavicular sonoanatomy must be correctly identified [40]. Moreover, despite the continuously growing evidence base, long-term comparative data are still not as prevalent as more proven methods of brachial plexus block [50].

In modern regional anesthesia practice, the costoclavicular brachial plexus block has a clearly defined role. At the level of the costoclavicular space, the lateral, medial, and posterior cords are clustered lateral to the axillary artery, and the musculocutaneous nerve has typically not yet exited the sheath, remaining in proximity to the lateral cord. This consistent anatomical arrangement facilitates reliable blockade of the musculocutaneous nerve with a single injection, reducing the need for separate supplementation that may be required with more distal approaches such as the axillary block. This characteristic contributes to its suitability for procedures involving the distal arm, elbow, forearm, and hand while maintaining a predictable block profile [41]. It is favourable to distal and intermediate upper limb surgery, to those patients in whom preserving the diaphragm is critically important, and in those settings, where procedural efficiency and safety are paramount [43]. The further development of comparative and outcome-based studies will iron out their suggestions and establish their place in the strategies of anesthesia based on anatomy [49]. Table 3 shows that the costoclavicular brachial plexus block occupies a defined, evidence-based role among contemporary regional anesthesia techniques.

**Table 3:** Clinical positioning of the costoclavicular brachial plexus block Compiled by the authors based on information synthesized from [31-34,37,39-41,43,45,49,50].

Evidence Domain	Finding	Clinical Interpretation	Reference
Comparative evidence	Randomized trials and meta-analyses define the role of the costoclavicular block.	Evidence-based alternative to conventional brachial plexus blocks	[34]
Surgical indications	Non-inferior success for distal and intermediate upper limb surgery	Suitable primary block for these procedures	[31]
Procedural performance	Similar or faster onset with fewer needle passes	Reflects efficient sonoanatomy and block dynamics	[33]
Technical advantages	Reproducible cord visualization and reduced needle depth	Improves technical reliability and consistency	[37]
Respiratory safety	Lower incidence of hemidiaphragmatic paralysis	Preferably in respiratory-vulnerable patients	[39]
Local anesthetic use	Effective blockade with moderate volumes	Enhances safety without compromising efficacy	[32]
Shoulder surgery limitation	Inadequate as a sole block for complete shoulder anesthesia	Requires supplementation or alternative approaches	[45]
Operator dependence	Performance is influenced by experience during early adoption	Highlights the importance of training and familiarity	[40]
Evidence gaps	Limited long-term comparative outcome data	Need for further high-quality studies	[50]
Role in current practice	Well-defined indications within anatomy-based anesthesia	Supports tailored patient- and procedure-specific use	[41]
Patient selection	Advantageous when diaphragmatic preservation is required	Optimises safety in high-risk populations	[43]
Future direction	Ongoing outcome-focused research	Expected refinement of indications	[49]

Summary of included key studies

Table 4 summarizes and categorizes key studies included in this narrative review.

**Table 4:** Summary of key articles on the costoclavicular brachial plexus block and related comparative evidence

Article	Category	Study type	Primary focus/comparator (from title)
Sala-Blanch X (2016) [39]	Anatomical/cadaveric foundation	Cadaver anatomic study	Anatomic basis for brachial plexus block at the costoclavicular space
Bailey JG (2021) [36]	Anatomical/cadaveric foundation	Cadaver study	Critical structures in the needle path of the costoclavicular block
Li JW (2017) [14]	Technique/sonoanatomy	Technique + sonoanatomy review	Sonoanatomy, technique, and block dynamics for costoclavicular block
Beh ZY (2017) [49]	Clinical application	Clinical report/series (vascular access context)	Costoclavicular approach for vascular access surgery
Silva GR (2019) [21]	Special populations	Clinical report/series	Costoclavicular block as an alternative in obese patients
Reeves MT (2023) [15]	Case report	Case report	Costoclavicular block for painless upper extremity reduction
Zhu M (2025) [6]	Case report/pregnancy	Case report + literature review	Continuous costoclavicular block for humeral fractures in pregnancy
Pirotesak S (2025) [1]	Review	Narrative review	Costoclavicular block for shoulder surgery
Xing T (2023) [2]	Review	Narrative review	Costoclavicular space approach narrative review
Tinoco J (2022) [3]	Review	Narrative review	Review of current evidence on costoclavicular block
Hsu AC (2019) [37]	Review/nomenclature	Comprehensive review	Infraclavicular block review using unified nomenclature
Brattwall M (2016) [19]	Review (regional anesthesia update)	Narrative update	Upper extremity nerve block benefit, duration, and safety update
Kaye AD (2021) [22]	Comparative overview	Narrative review	Supraclavicular vs infraclavicular blocks: considerations
Amaral S (2024) [4]	Evidence synthesis	Systematic review + meta-analysis	Infraclavicular vs costoclavicular approaches
Garg H (2024) [11]	Evidence synthesis	Systematic review + meta-analysis	Classical infraclavicular vs costoclavicular approach
Casas-Arroyave FD (2021) [7]	Evidence synthesis	Systematic review + meta-analysis	Complications across three brachial plexus block techniques
Koo CH (2023) [9]	Evidence synthesis (safety)	Meta-analysis	Hemidiaphragmatic paralysis after costoclavicular vs other blocks
Muir D (2024) [8]	Evidence synthesis (comparative)	Systematic review + meta-analysis of RCTs	Supraclavicular vs infraclavicular blocks in orthopedic surgery
Nijs K (2024) [10]	Evidence synthesis (comparative)	Systematic review + meta-analysis	Axillary block vs other techniques in distal upper limb surgery
Wu L (2023) [32]	Evidence synthesis (local anesthetic)	Systematic review + meta-analysis	Optimal ropivacaine concentration for brachial plexus blocks
Leurcharusmee P (2017) [18]	Randomized trials (approach comparison)	Randomized trial	Costoclavicular vs paracoracoid infraclavicular block
Kavrut Ozturk N (2017) [24]	Randomized/controlled comparative	Comparative study	Coracoid vs retroclavicular infraclavicular approaches
Songthamwat B (2018) [33]	Randomized trials (approach comparison)	Prospective randomized comparison	Lateral sagittal vs costoclavicular infraclavicular approach
Dost B (2021) [45]	Randomized trials (block dynamics)	Randomized clinical trial	Lateral sagittal vs costoclavicular: block dynamics
Cesur S (2021) [27]	Randomized trials (approach comparison)	Randomized comparison	Costoclavicular vs infraclavicular block
Lee MG (2020) [35]	Randomized trials (injection strategy)	Randomized controlled trial	Single vs triple injection using costoclavicular approach
Luo Q (2020) [50]	Randomized trials (comparative technique)	Randomized non-inferiority trial	Supraclavicular vs costoclavicular with modified double-injection
Wang S (2023) [23]	Randomized trials (dose/concentration)	Randomized double-blind noninferiority trial	0.5% vs 0.375% ropivacaine for costoclavicular block
Luo Q (2023) [38]	Randomized trials (shoulder surgery comparator)	Randomized prospective non-inferiority study	Costoclavicular vs interscalene for arthroscopic shoulder surgery
Guzel M (2023) [17]	Randomized trials (pediatric)	Randomized clinical trial	Supraclavicular vs costoclavicular blocks in pediatric patients
Nalini KB (2021) [16]	Randomized trials (comparative block)	Randomized clinical study	Costoclavicular vs axillary block
Zhang L (2021) [20]	Observational comparative	Propensity score-matched retrospective cohort	Costoclavicular vs supraclavicular block
Soylu S (2024) [34]	Randomized trials (adjunct)	Randomized controlled trial	Neurostimulator usage effect on costoclavicular block success
Dhanger S (2025) [12]	Randomized trials (approach comparison)	Randomized comparison	Costoclavicular vs paracoracoid approaches
Rao A (2025) [25]	Randomized trials (approach comparison)	Prospective randomized interventional study	Lateral sagittal vs costoclavicular approaches
Senapati LK (2025) [47]	Randomized trials (comparative block)	Randomized controlled trial	Costoclavicular vs supraclavicular block
Baral U (2025) [30]	Randomized trials (distal surgery comparator)	Randomized controlled study	Costoclavicular vs axillary approach in wrist/hand surgery
Ram K (2025) [29]	Randomized trials (adjuvant)	Randomized study	Levobupivacaine ± dexamethasone for costoclavicular block
Wang J (2025) [28]	Protocol	Dose-finding protocol	Weight-adjusted effective volume protocol for combined blocks in shoulder arthroscopy
La Palma J (2025) [48]	Pediatric safety	Incidence study	Phrenic nerve palsy in pediatric costoclavicular block
Duplechin DP (2025) [5]	Review (related approach)	Narrative review	Retroclavicular block efficacy and safety after shoulder surgeries
Zhuo Q (2022) [43]	Adjacent/related regional technique	Prospective randomized trial	Clavipectoral fascial plane block + cervical plexus block for clavicle surgery
Gupta S (2025) [26]	Adjacent/related regional technique	Double-blind randomized trial	Supraclavicular + clavipectoral fascial plane vs SCUT block in clavicle surgery
Ali B (2024) [46]	Special populations	Secondary analysis of RCT	Brachial plexus blocks in obesity
Li F (2023) [44]	Pediatric adjunct anesthesia	Retrospective study	Brachial plexus block with GA in pediatric elbow fracture context

Limitations and future directions

This review was intentionally designed as a narrative synthesis rather than a systematic review or meta-analysis. The current body of literature on the costoclavicular brachial plexus block is methodologically heterogeneous, encompassing cadaveric anatomical studies, imaging-based technical descriptions, small randomized trials with differing primary endpoints, observational cohorts, case reports, and comparative studies using non-uniform outcome measures. Substantial variability exists in block techniques (single vs multiple injections, needle trajectories), surgical indications (shoulder, forearm, distal limb procedures), anesthetic concentrations and volumes, and definitions of block success, onset time, and complications. Such clinical and methodological diversity limits the validity of quantitative pooling and risks producing misleading summary estimates. Furthermore, several key contributions to the field are foundational anatomical and technical investigations that are not amenable to meta-analytic aggregation but are essential for understanding block performance and safety. In this context, a narrative framework was considered more appropriate to integrate mechanistic insights, procedural nuances, and evolving clinical evidence into a coherent and clinically interpretable overview. The absence of a formal systematic methodology may introduce selection bias and limit the ability to provide pooled effect estimates, which should be addressed in future high-quality systematic reviews once greater methodological uniformity emerges in the literature.

## Conclusions

This narrative review integrates anatomical, sonoanatomical, and clinical evidence to evaluate the safety and effectiveness of the costoclavicular brachial plexus block for upper limb surgery. Current data support its reliability in providing surgical anesthesia and effective postoperative analgesia for distal and mid-upper limb procedures, with high success rates under ultrasound guidance. The compact arrangement of the cords within the costoclavicular space facilitates efficient local anesthetic spread and supports both single-injection and continuous catheter techniques, while demonstrating lower rates of hemidiaphragmatic paralysis compared with interscalene and supraclavicular approaches. Although its role in extensive shoulder surgery remains limited, the costoclavicular approach represents a valuable and increasingly established technique in contemporary regional anesthesia practice, warranting further standardization and multicenter investigation.
